# Succinate of Ammonia—a Remedy for Delirium Tremens

**Published:** 1845-01

**Authors:** 


*Succinate of Ammonia—a remedy for Delirium Tremens.*—M. Scharn has successfully employed the succinate of ammonia for delirium tremens. The most furious delirium is quieted by the remedy as if by magic, and the disease cured by it in a few hours without the aid of any other medicine.—*Jour. de Pharm.*, from *American Journal.*

